# SIRT1 genetic variants associate with the metabolic response of Caucasians to a controlled lifestyle intervention – the TULIP Study

**DOI:** 10.1186/1471-2350-9-100

**Published:** 2008-11-12

**Authors:** Peter Weyrich, Fausto Machicao, Julia Reinhardt, Jürgen Machann, Fritz Schick, Otto Tschritter, Norbert Stefan, Andreas Fritsche, Hans-Ulrich Häring

**Affiliations:** 1 Department of Internal Medicine, Division of Endocrinology, Diabetology, Vascular Disease, Nephrology and Clinical Chemistry, University of Tübingen, Germany; 2 Section on Experimental Radiology, University of Tübingen, Germany

## Abstract

**Background:**

Sirtuin1 (SIRT1) regulates gene expression in distinct metabolic pathways and mediates beneficial effects of caloric restriction in animal models. In humans, *SIRT1 *genetic variants associate with fasting energy expenditure. To investigate the relevance of SIRT1 for human metabolism and caloric restriction, we analyzed *SIRT1 *genetic variants in respect to the outcome of a controlled lifestyle intervention in Caucasians at risk for type 2 diabetes.

**Methods:**

A total of 1013 non-diabetic Caucasians from the Tuebingen Family Study (TUEF) were genotyped for four tagging *SIRT1 *SNPs (rs730821, rs12413112, rs7069102, rs2273773) for cross-sectional association analyses with prediabetic traits. SNPs that associated with basal energy expenditure in the TUEF cohort were additionally analyzed in 196 individuals who underwent a controlled lifestyle intervention (Tuebingen Lifestyle Intervention Program; TULIP). Multivariate regressions analyses with adjustment for relevant covariates were performed to detect associations of *SIRT1 *variants with the changes in anthropometrics, weight, body fat or metabolic characteristics (blood glucose, insulin sensitivity, insulin secretion and liver fat, measured by magnetic resonance techniques) after the 9-month follow-up test in the TULIP study.

**Results:**

Minor allele (X/A) carriers of rs12413112 (G/A) had a significantly lower basal energy expenditure (*p *= 0.04) and an increased respiratory quotient (*p *= 0.02). This group (rs12413112: X/A) was resistant against lifestyle-induced improvement of fasting plasma glucose (GG: -2.01%, X/A: 0.53%; *p *= 0.04), had less increase in insulin sensitivity (GG: 17.3%, X/A: 9.6%; *p *= 0.05) and an attenuated decline in liver fat (GG: -38.4%, X/A: -7.5%; *p *= 0.01).

**Conclusion:**

*SIRT1 *plays a role for the individual lifestyle intervention response, possibly owing to decreased basal energy expenditure and a lower lipid-oxidation rate in rs12413112 X/A allele carriers. *SIRT1 *genetic variants may, therefore, represent a relevant determinant for the response rate of individuals undergoing caloric restriction and increased physical activity.

## Background

The sirtuin SIRT1 (silent mating type information regulation 2 homolog 1) is a NAD^+^-dependent deacetylase involved in regulation of glucose metabolism in different organs [1,2]. SIRT1 directs its deacetylase activity against histones, a process that facilitates the formation of heterochromatin with subsequent repression of gene transcription. In addition, SIRT1 directly regulates activity of metabolic transcription factors and their co-activators, as e. g. forkhead transcription factor FOXO1, nuclear factor NF-κB (nuclear factor of kappa light polypeptide gene enhancer in B-cells 1) or PGC-1α (peroxisome proliferator-activated receptor gamma coactivator 1 alpha).

Due to its ubiquitous expression, SIRT1 activity is relevant for many insulin-sensitive organs. In adipose tissue, SIRT1 down-regulates fat storage by increased lipolysis via the inactivation of PPARγ (peroxisome proliferator-activated receptor gamma) [3]. In pancreatic β-cells, SIRT1 enhances glucose-stimulated insulin secretion via down-regulation of UCP2 (uncoupling protein 2) [4]. In the liver, particularly under low-nutrient conditions, SIRT1 induces gluconeogenesis and inhibits glycolysis by deacetylating PGC-1α [5]. In skeletal muscle of obese mice, SIRT1 mediates the insulin sensitizing effect of resveratrol. The antioxidant resveratrol induces increased exercise endurance and higher basal energy expenditure in mice, thereby promoting resistance against diet-induced obesity [6] and mortality [7]. Interestingly, modest overexpression of SIRT1 also protects mice against high-fat diet induced glucose intolerance and hepatic steatosis [8], so that SIRT1 may represent a promising future pharmacological target to prevent the metabolic sequelae of chronic exposure to a high-fat diet.

Summarizing all these data, one may assume that human SIRT1 genetic variants may also play a role in lifestyle intervention response in humans. So far, no studies exist on whether *SIRT1 *genetic variants affect the individual metabolic susceptibility to caloric restriction or increased physical activity. The only genetic study that assessed *SIRT1 *genetic variants in the context of metabolism and diabetes reports an association of *SIRT1 *genetics with basal energy expenditure in Finnish type 2 diabetic patients [6]. We, therefore, conducted the present study to investigate *SIRT1 *variants in the context of a controlled lifestyle intervention in prediabetic individuals at high risk for later onset of diabetes. For this purpose, 1013 subjects from the cross-sectional Tuebingen Family Study (TUEF) were genotyped for *SIRT1 *tagging SNPs and association analyses for energy expenditure and other metabolic traits were undertaken. In a second step, genotypes that associated with different phenotypes in the TUEF cohort were analyzed in the ongoing Tübingen Lifestyle Intervention Programme (TULIP) [9] for longitudinal changes of blood glucose, insulin secretion, insulin sensitivity and liver fat.

## Methods

### Study design and participants

All volunteers signed written consent to the study protocol, approved by the local medical ethics committee. Genotyping for *SIRT1 *was done in 1013 study participants, 96 of which were excluded (manifest diabetes or missing data), resulting in a total cohort of n = 917 for cross-sectional analyses. 196 participants of the study cohort also participated in TULIP, so that longitudinal (9 month) data on metabolic traits including liver fat content were available. TULIP participants are at increased risk of type 2 diabetes (family history of type 2 diabetes, body mass index (BMI) > 27 kg/m^2^, diagnosis of IGT or previous diagnosis of gestational diabetes) and undergo a controlled lifestyle intervention designed for the same targets as the Diabetes Prevention Study [10]: body weight reduction by > 5%, dietary fat (saturated fatty acids) reduction to < 30% (< 10%) of caloric intake, increase of dietary fibre (> 15 g/1000 kcal) and enhanced physical activity (> 3 h/week; see [9] for further details on TULIP).

### Oral glucose tolerance test (OGTT)

All participants underwent an OGTT performed according to WHO recommendations [11]. Blood glucose, plasma insulin, plasma C-peptide and non-esterified fatty acid (NEFA) levels were determined at 0, 30, 60, 90 and 120 min intervals.

### Insulin sensitivity, insulin secretion, and analytical methods

Blood glucose was determined using a bedside glucose analyzer (Yellow Springs Instruments, Yellow Springs, CO, USA). Plasma insulin was determined by microparticle enzyme immunoassay (Abbott Laboratories, Tokyo, Japan), and plasma C-peptide was determined by radioimmunoassay (Byk-Sangtec, Dietzenbach, Germany). Insulin sensitivity was estimated using the model from Matsuda and DeFronzo [12], and insulin secretion was estimated from 30 min C-peptide levels obtained during the OGTT. Basal energy expenditure and respiratory quotient were measured with a DELTATRAC™ Metabolic Monitor (Hoyer; Bremen, Germany) under resting conditions. Further details on analytical procedures are provided elsewhere [13].

### Determination of body and liver fat

Total body fat and lean body mass were measured by bioelectrical impedance (RJL; Detroit, MI, USA). Liver fat was determined by magnetic resonance spectroscopy (1.5T Magnetom Sonata; Siemens, Erlangen, Germany) in the posterior 7^th ^segment of the liver. A single-voxel stimulated echo acquisition mode technique was applied (repetition time = 4 s, echo time = 10 msec, 32 acquisitions) for a voxel of 3 × 3 × 2 cm^3^, and liver fat content was calculated by the signal integral (methylene/methyl signals between 0.7–1.5 ppm) in reference to the sum of water and lipid signal integrals [14].

### Genotyping

The TaqMan assay (Eurogentec; Liege, Belgium) and the fluorescence detecting ABI Prism 7500 (Applied Biosystems; Foster City, CA, USA) were used for genotyping of SNPs selected by HapMap analysis (phase I, release 21a, January 2007). A minor allele frequency (MAF) > 0.05 and a linkage disequilibrium measure (r^2^) < 0.8 were prerequisites for tagging SNP selection in a region comprising the whole *SIRT1 *gene and 10 kb of its promoter. To monitor TaqMan test reproducibility and accuracy, samples of 50 individuals were sequenced for the selected tagging SNPs with an ABI Prism 310 genetic analyser (Applied Biosystems). Subsequently, one sample per allelic combination was included in each TaqMan assay as a known reference genotype control.

### Statistics

Data are presented as means ± SD. The Hardy-Weinberg equilibrium was calculated by the χ^2^-test. Multivariate linear regression analyses with adjustments for relevant covariates were undertaken, using logarithmically transformed data for non-normally distributed parameters. The JMP 4.0.4 software (SAS Institute; Cary, NC, USA) was used for statistical analyses, and *p *values ≤ 0.05 were considered statistically significant.

## Results

### *SIRT1 *genetic variants in the TUEF/TULIP cohort

Four SNPS of the *SIRT1 *gene (~35 kb, 9 exons) on chromosome 10q21.3 were selected from the HapMap according the selection criteria for genotyping: rs730821 (A/G, MAF = 0.186 in the TUEF cohort, promoter), rs12413112 (G/A, MAF = 0.114, intron4), rs7069102 (G/C, MAF = 0.301, intron4) and rs2273773 (T/C, MAF = 0.058, intron5). All tagging SNPs were in Hardy-Weinberg-Equilibrium (*p *> 0.21; all, both TUEF and TULIP cohort), and linkage disequilibrium (r^2^) ranged from 0.008 to 0.53.

### Cross-sectional TUEF study

We first verified whether *SIRT1 *genetic variants associate with basal energy expenditure in the TUEF cohort, as shown before in a Finnish population [6]. Indeed, basal energy expenditure was significantly lower in rs12413112 (*p *= 0.04; dominant model) and rs7069102 (*p *= 0.05; dominant model) minor allele carriers compared to homozygous carriers of the major G alleles, after adjustment for relevant covariates as age, sex, lean body mass and body fat [15-17]. Minor allele carriers of rs12413112, in addition, had a significantly increased respiratory quotient (RQ; +2.2%, *p *= 0.02; dominant model; see Table 1). In contrast, anthropometrics (BMI, body fat, waist-to-hip ratio) did not differ among *SIRT1 *genotypes. There was no significant association with (fasting and 2 h OGTT) glucose and insulin plasma levels, insulin sensitivity (OGTT and clamped) or insulin secretion (dominant *p *for all > 0.12; Table 1). Serum lipid parameters (total-, HDL-, LDL-cholesterol, apolipoproteins, NEFAs) did also not differ between *SIRT1 *genotypes (data not shown).

**Table 1:** Distribution of anthropometrics and metabolic traits in the TUEF cohort according to rs12413112, rs7069102, rs730821 and rs2273773 in *SIRT1*

SNP rs12413112	Genotype			Calculation Model		
		
	GG	GA	AA	p^add^	p^dom^	p^rec^
n	726	173	18			
Sex (men/women), *n*	266/460	72/101	6/12	0.45^a^	0.29	0.71
Age (years)	38.6 ± 12.5	38.9 ± 12.8	37.9 ± 13.9	0.92	0.86	0.71
Waist-to-hip ratio	0.87 ± 0.09	0.87 ± 0.09	0.86 ± 0.12	0.69^b^	0.39	0.80
BMI (kg/m^2^)	29.3 ± 8.8	29.1 ± 8.0	29.7 ± 8.7	0.08	0.46	**0.02**
Body fat (%)	30.4 ± 10.9	30.1 ± 11.2	34.0 ± 10.7	0.29	0.53	0.12
Fasting glucose (mmol/l)	5.12 ± 0.66	5.13 ± 0.62	5.07 ± 0.55	0.87^c^	0.83	0.61
2 h glucose (mmol/l)	6.27 ± 1.73	6.42 ± 1.62	5.84 ± 1.79	0.07	0.42	0.08
Fasting insulin (pmol/l)	64.8 ± 55.1	61.6 ± 47.0	63.5 ± 57.5	0.52	0.35	0.26
2 h insulin (pmol/l)	429 ± 442	441 ± 366	397 ± 337	0.09	0.42	0.29
Insulin sensitivity (AU)	16.6 ± 11.0	15.8 ± 10.7	16.6 ± 8.9	0.19	0.90	0.19
Insulin secretion (pmol/l)	2046 ± 898	2115 ± 907	2172 ± 1054	0.68	0.49	0.53
Basal energy exp (kcal/24 h)^d^	1730 ± 297	1746 ± 331	1655 ± 292	0.10	**0.039**	0.31
Respiratory quotient^d^	0.81 ± 0.07	0.83 ± 0.07	0.81 ± 0.08	**0.048**	**0.017**	0.92
rs7069102	GG	GC	CC			

n	457	366	94			
Sex (men/women), *n*	172/285	138/228	34/60	0.96	0.94	0.78
Age (years)	38.2 ± 12.2	39.4 ± 12.8	37.9 ± 13.1	0.36	0.26	0.48
Waist-to-hip ratio	0.86 ± 0.09	0.87 ± 0.09	0.86 ± 0.10	0.84	0.92	0.56
BMI (kg/m^2^)	29.5 ± 8.9	29.1 ± 8.7	28.8 ± 7.5	0.51	0.48	0.53
Body fat (%)	30.5 ± 11.2	30.2 ± 10.7	30.6 ± 11.4	0.92	0.68	0.88
Fasting glucose (mmol/l)	5.13 ± 0.68	5.12 ± 0.63	5.08 ± 0.55	0.79	0.64	0.65
2 h glucose (mmol/l)	6.27 ± 1.74	6.30 ± 1.64	6.32 ± 1.86	0.94	0.89	0.73
Fasting insulin (pmol/l)	65.2 ± 51.6	63.2 ± 56.6	62.3 ± 52.1	0.73	0.92	0.43
2 h insulin (pmol/l)	441 ± 450	418 ± 401	432 ± 408	0.99	0.89	0.95
Insulin sensitivity (AU)	16.7 ± 11.6	16.2 ± 10.4	16.2 ± 9.9	0.88	0.70	0.67
Insulin secretion (pmol/l)	2091 ± 947	2017 ± 856	2097 ± 866	0.47	0.44	0.44
Basal energy exp (kcal/24 h)	1742 ± 288	1734 ± 334	1680 ± 245	0.06	**0.05**	0.56
Respiratory quotient	0.81 ± 0.07	0.82 ± 0.07	0.81 ± 0.07	0.35	0.18	0.90
rs730821	AA	AG	GG			

n	603	286	28			
Sex (men/women), *n*	237/366	94/192	13/15	0.11	0.12	0.33
Age (years)	38.4 ± 12.4	39.3 ± 12.9	37.5 ± 13.4	0.59	0.37	0.58
Waist-to-hip ratio	0.87 ± 0.09	0.87 ± 0.09	0.86 ± 0.12	0.38	0.55	0.28
BMI (kg/m^2^)	29.5 ± 8.7	29.0 ± 8.6	27.7 ± 7.5	0.28	0.12	0.39
Body fat (%)	30.4 ± 11.2	30.5 ± 10.4	27.2 ± 11.3	0.23	0.26	0.13
Fasting glucose (mmol/l)	5.13 ± 0.67	5.11 ± 0.59	5.01 ± 0.66	0.39	0.66	0.22
2 h glucose (mmol/l)	6.28 ± 1.72	6.31 ± 1.66	6.11 ± 2.10	0.34	0.92	0.14
Fasting insulin (pmol/l)	65.1 ± 51.4	61.7 ± 57.5	68.8 ± 61.8	0.55	0.92	0.68
2 h insulin (pmol/l)	443 ± 440	406 ± 387	433 ± 527	0.62	0.69	0.55
Insulin sensitivity (AU)	16.5 ± 11.5	16.2 ± 9.6	18.1 ± 12.3	0.80	0.82	0.91
Insulin secretion (pmol/l)	2098 ± 945	1997 ± 822	1929 ± 694	0.68	0.38	0.64
Basal energy exp (kcal/24 h)	1746 ± 305	1714 ± 306	1642 ± 217	0.89	0.86	0.63
Respiratory quotient	0.82 ± 0.07	0.81 ± 0.07	0.82 ± 0.08	0.79	0.48	0.88
rs2273773	TT	TC	CC			

n	812	103	2			
Sex (men/women), *n*	313/499	30/73	1/1	0.16	0.07	0.72
Age (years)	38.7 ± 12.4	38.5 ± 13.6	33.0 ± 4.2	0.82	0.80	0.66
Waist-to-hip ratio	0.87 ± 0.09	0.86 ± 0.10	0.77 ± 0.02	0.10	0.68	**0.04**
BMI (kg/m^2^)	29.3 ± 8.9	29.2 ± 7.2	24.3 ± 7.1	0.72	0.99	0.42
Body fat (%)	30.2 ± 11.1	31.9 ± 9.7	28.0 ± 18.4	0.78	0.52	0.84
Fasting glucose (mmol/l)	5.13 ± 0.65	5.05 ± 0.62	4.96 ± 0.09	0.19	0.35	0.93
2 h glucose (mmol/l)	6.27 ± 1.71	6.47 ± 1.75	4.58 ± 0.35	0.39	0.31	0.18
Fasting insulin (pmol/l)	63.4 ± 50.5	70.2 ± 74.3	33.0 ± 22.6	0.44	0.61	0.22
2 h insulin (pmol/l)	426 ± 417	470 ± 494	178 ± 212	0.17	0.71	0.06
Insulin sensitivity (AU)	16.5 ± 11.0	15.9 ± 10.1	26.7 ± 14.1	0.38	0.82	0.21
Insulin secretion (pmol/l)	2070 ± 908	2019 ± 864	1299 ± 41	0.45	0.34	0.39
Basal energy exp (kcal/24 h)	1730 ± 304	1756 ± 310	1835 ± 0	0.33	0.16	0.51
Respiratory quotient	0.82 ± 0.07	0.80 ± 0.08	0.84 ± 0	0.19	0.09	0.94

Data are presented as means ± SD; AU, arbitrary units

^a^According to χ^2 ^test; ^b^Waist-to-hip ratio, body mass index (BMI) and body fat were adjusted for age and sex; ^c^All the following parameters were adjusted for age, sex and body fat; insulin secretion was additionally adjusted for insulin sensitivity; ^d^*n *= 375 participants; basal energy expenditure was adjusted to age, sex, lean body mass and body fat.

### *SIRT1 *variants and TULIP lifestyle intervention outcome traits

The two *SIRT1 *SNPs that associated with energy expenditure, namely rs12413112 and rs7069102, were subsequently analyzed in 196 individuals (118 females, 78 males, aged 45.8 ± 11.2 years; GG: n = 151, GA: n = 42, AA: n = 3 individuals) from the TULIP study cohort who had completed the 9-month follow-up test. In this cohort, average weight loss amounted to 2.56 ± 4.06 kg in a follow-up time of 259 ± 53 days, resulting in a BMI reduction of 0.87 ± 1.38.

**Figure 1:**
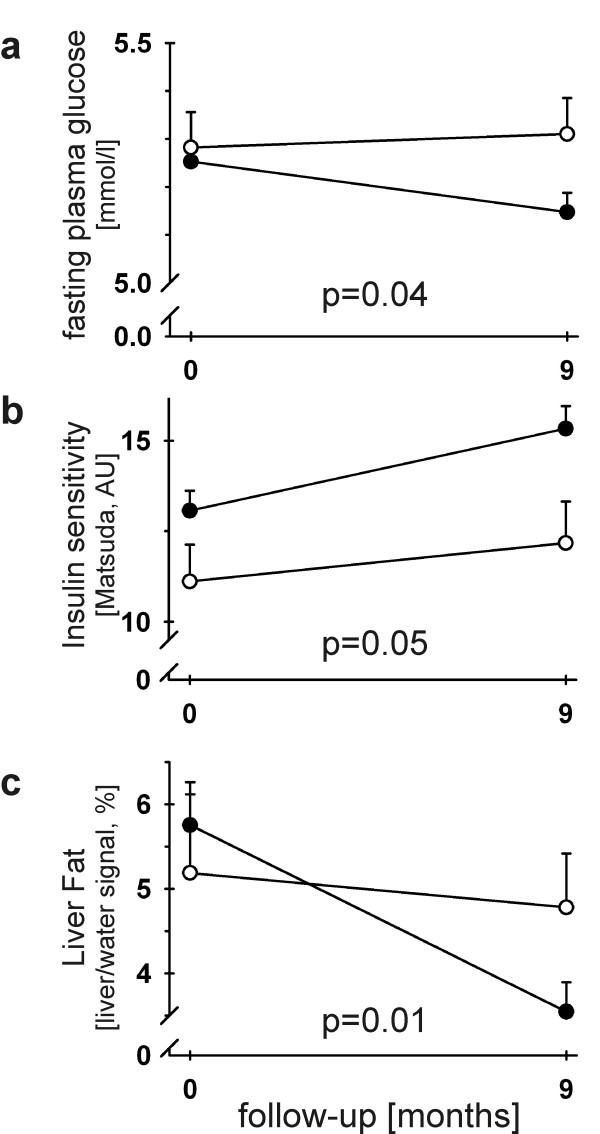
**Interaction of rs12413112 *SIRT1 *genotype with the metabolic responses during a controlled 9-month lifestyle intervention**. Rs12413112 *SIRT1 *genotype (GG): filled circles (●); X/A: opened circles (○). a) Response of plasma glucose determined during the fasting state. b) Response of insulin sensitivity estimated by the formula from Matsuda et al. [12]. c) Response of liver fat content, determined by magnetic resonance spectroscopy. For statistical analyses, all data were log-transformed and adjusted for age, sex, follow-up-time, BMI at both baseline and follow-up and the corresponding variable (plasma glucose, insulin sensitivity or liver fat) measured at baseline. Data are presented as means+SEM (G/G: n = 152, X/A: n = 45 TULIP participants).

We did not detect significant associations between rs12413112 genotype and weight (*p *= 0.37) or BMI (*p *= 0.38) change. By contrast, minor allele carriers of rs12413112 were resistant against lifestyle-induced improvement of fasting plasma glucose (GG: -2.01%, X/A: 0.53%; *p *= 0.04, adjusted for age, sex, follow-up time, BMI at baseline and follow-up and fasting plasma glucose at baseline; Fig. 1A), had a weaker increase in insulin sensitivity (GG: 17.3%, X/A: 9.6%; *p *= 0.05, adjusted for age, sex, follow-up time, BMI at baseline and follow-up and insulin sensitivity at baseline; Fig. 1B) and a remarkably attenuated decline in liver fat content (GG: -38.4%, X/A: -7.5%; *p *= 0.01, adjusted for age, sex, follow-up time, BMI at baseline and follow-up and liver fat content at baseline; Fig. 1C). The effect on liver fat content remained significant even after Bonferroni's correction for multiple testing (2 tests: energy expenditure and obesity), and we had a power of 0.76 to detect this effect. The rs12413112 genotype effects on fasting glucose (0.50) and insulin sensitivity (0.56) change did not show a sufficient statistical power and also did not remain significant after correction for multiple comparisons. The rs7069102 genotype did not significantly associate with longitudinal changes in weight (p = 0.40; dominant model), BMI (p = 0.41), fasting glucose (p = 0.08), insulin sensitivity (p = 0.45) and liver fat (p = 0.09) in the longitudinal TULIP analysis.

## Discussion

This is the first study to have analysed the impact of *SIRT1 *genetic variants on the outcome of a lifestyle intervention in a diabetes risk population. Despite numerous works demonstrating *SIRT1 *function in different insulin-responsive tissues [1,3-5,18,19], we were not able to verify a significant association of *SIRT1 *variants with plasma glucose, plasma insulin, NEFAs, serum lipids, insulin sensitivity or insulin secretion, both under fasting conditions and 2 h OGTT in the cross-sectional analyses. However, minor allele carriers of rs12413112 had significantly lower basal energy expenditure and an increased respiratory quotient (RQ) at baseline. Interestingly, in a Finnish population, higher basal energy expenditure was reported for three *SIRT1 *SNPs [6]. This difference may be due to the very low linkage disequilibrium of these three SNPs with rs12413112, ranging from 0.0076 to 0.10 in both the TUEF cohort as well as the CEU population of the HapMap project, so that rs12413112 may act in the opposing direction on SIRT1 function compared to the previously reported SNPs. In addition, our study cohort has considerably differing allele frequencies compared to the investigated Finnish population, at least in the *SIRT1 *locus.

The minor allele of rs12413112 is associated with unresponsiveness of fasting plasma glucose, insulin sensitivity and liver fat to a controlled 9 month lifestyle intervention. Because of the higher RQ in this subgroup, one may speculate that the rs12413112 minor A-allele contributes to a lower rate of lipid-oxidation, resulting in the observed lower liver fat reduction, despite dietary changes and increased physical activity. Taking into account that modest overexpression of SIRT1 leads to protection from high-fat diet induced hepatic steatosis in mice [8], our study let assume that the minor allele of rs12413112 may negatively affect *SIRT1 *expression, especially as the SNPs tagged by this intronic SNP, namely rs11599151, rs11599524 and rs12778366, are all located in the *SIRT1 *promoter.

Finally, the rather small study sample size has to be mentioned as the major statistical limitation of this study. The here reported effects of *SIRT1 *variants are statistically significant, but a conservative adjustment for multiple comparisons would render the associations in TUEF with energy expenditure and RQ non-significant. However, we think that our findings are relevant, as the TULIP study provides a very precisely phenotyped and closely monitored lifestyle intervention cohort which is suitable for studying the role of genetic variants by a classical candidate-gene approach.

## Conclusion

In summary, our study shows that *SIRT1 *genetic variants may affect individual susceptibility to caloric restriction and increased physical activity. Although the cross-sectional study let rather doubt that *SIRT1 *does represent a classical diabetes risk gene, *SIRT1 *genetic variants may determine the individual response to a lifestyle intervention. Beyond *AdipoR1 *[14] and *PPARδ *[20], *SIRT1 *is the third gene detected which does not affect prediabetic traits in a cross-sectional study approach, but which remarkably influences the response to a lifestyle intervention. Therefore, *SIRT1 *should be elucidated in other prospective lifestyle intervention studies, and further research on SIRT1 as a potential pharmacological target for prevention of type 2 diabetes is warranted.

## Competing interests

The authors declare that they have no competing interests.

## Authors' contributions

PW was responsible for the complete statistical analysis and prepared all tables, figures and the manuscript. FM is responsible of the genotyping facility at the University of Tübingen. JR made contributions to phenotyping of TULIP participants. JM and FS performed the magnetic resonance spectroscopy studies for liver fat determination. OT contributed to the statistical analysis. NS, AF and HUH acted as principal investigators for the TUEF and TULIP study, and were responsible for patient and data management. All authors contributed to the preparation of the manuscript.

## Pre-publication history

The pre-publication history for this paper can be accessed here:


